# Supplementary material for: A comprehensive proteome and the first phosphoproteome reveal extensive phosphorylation of carbohydrate metabolism in Cryptosporidium parvum sporozoites
Source: Parasit Vectors. 2026 Jul 2;19:408. doi: 10.1186/s13071-026-07538-3 (PMC13599311; doi:10.1186/s13071-026-07538-3)

Supporting Informaion, S1 Figure

Figure S1. Simplified reconstruction of carbohydrate metabolism in excysted *C. parvum* sporozoites. This schematic summarizes the major glycolytic and connected carbohydrate metabolic pathways identified in this study. Enzymes and pathways are shown for conceptual clarity and do not reflect quantitative protein abundance or phosphorylation levels, which are presented in Figures 7 and 8.

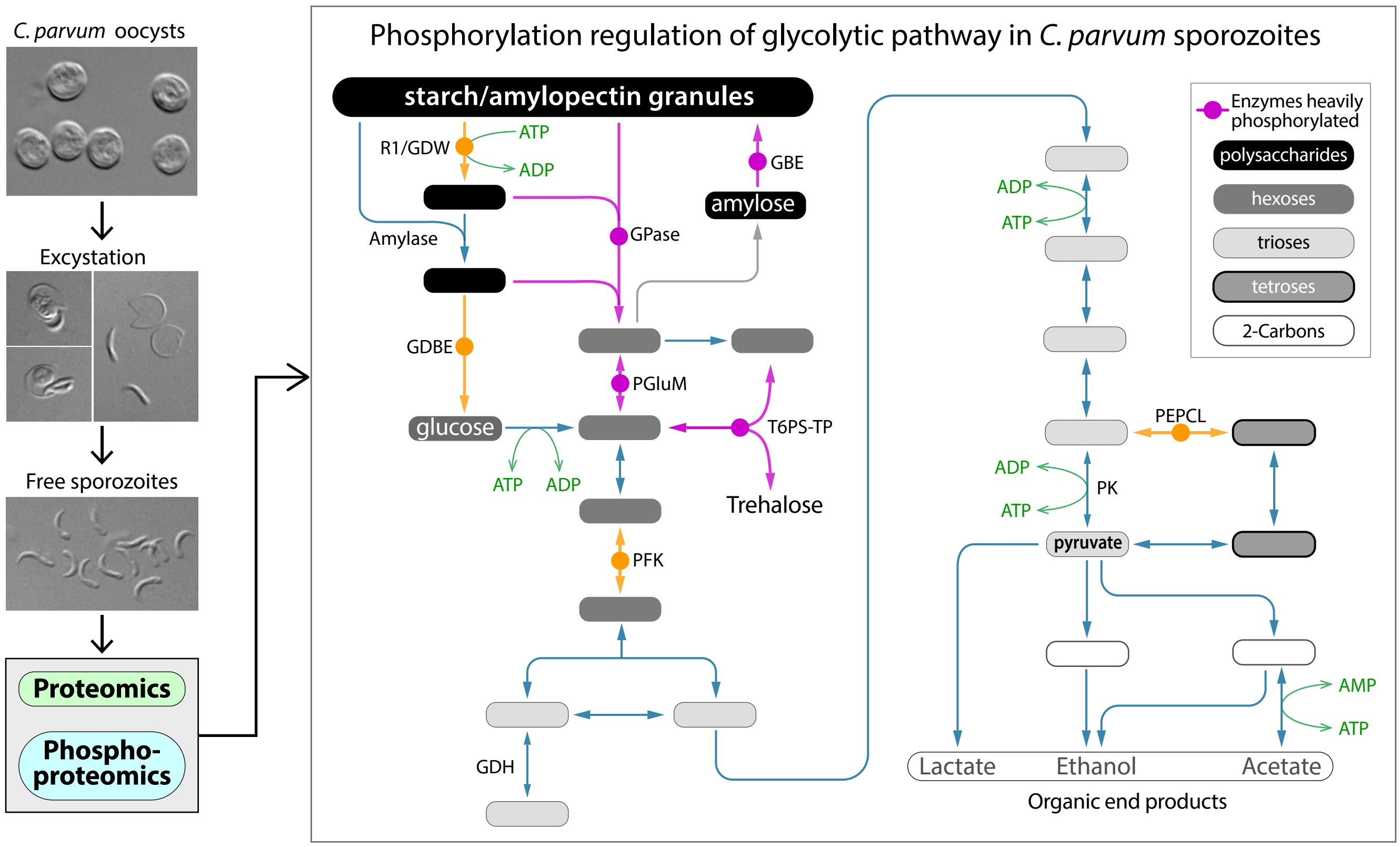

Supplement: Supplementary file 1 [file 13071_2026_7538_MOESM1_ESM.pdf]
